# Relationship between Texture, Hydrogen Content, Residual Stress and Corrosion Resistance of Electrodeposited Chromium Coating: Influence of Heat Treatment

**DOI:** 10.3390/ma17164142

**Published:** 2024-08-21

**Authors:** Jinghan Yang, Pengfei Ji, Xuemei Yang, Linyang Wu, Xiaoyun Ding, Jin Zhang, Yong Lian, Shitao Dou, Liming Jiang, Biliang Zhang

**Affiliations:** 1 Institute for Advanced Materials and Technology, University of Science and Technology Beijing, Beijing 100083, China; y1055865834@163.com (J.Y.); yangxuemei2021666@163.com (X.Y.); linyangwu2022@163.com (L.W.); dingxiaoyun@xs.ustb.edu.cn (X.D.); doushitao@126.com (S.D.); 2 Beijing Key Laboratory of Corrosion, Erosion and Surface Technology, University of Science and Technology Beijing, Beijing 100083, China; 3 National Materials Corrosion and Protection Data Center, University of Science and Technology Beijing, Beijing 100083, China; 4 Institute of Engineering Technology, University of Science and Technology Beijing, Beijing 100083, China; jipengfei@ustb.edu.cn; 5 Manufacturing Technology Department, Chongqing Jianshe Industry (Group) Co., Ltd., Chongqing 400054, China; 13136676733@163.com (L.J.); 19304636379@163.com (B.Z.)

**Keywords:** electrodeposited chromium coating, heat treatment, texture, hydrogen content, residual stress, corrosion resistance

## Abstract

Electrodeposited chromium plating continues to be widely used in a number of specialized areas, such as weapons, transport, aerospace, etc. However, the formation of texture, hydrogen content and residual stress can degrade the serviceability and lead to material failure. The effect of post heat treatment processes on the relationship of texture, hydrogen content, residual stress and corrosion resistance of hexavalent [Cr(VI)] chromium coatings deposited on Cr–Ni–Mo–V steel substrates was investigated. Macrotexture was measured by XRD. Microtexture, dislocation density and grain size were studied by EBSD. With the increase of the heat treatment temperature, it was found that the fiber texture strength of the (222) plane tended to increase and subsequently decrease. Below 600 °C, the increase in the (222) plane texture carried a decrease in the hydrogen content, residual stress, microhardness and an increase in the corrosion resistance. In addition, crack density and texture strength were less affected by the heat treatment time. Notably, relatively fewer crack densities of 219/cm^2^, a lower corrosion current density of 1.798 × 10^−6^ A/dm^2^ and a higher microhardness of 865 HV were found under the preferred heat treatment temperature and time of 380 °C and 4 h, respectively. The hydrogen content and residual stress were 7.63 ppm and 61 MPa, with 86% and 75% reduction rates compared to the as-plated state, respectively. In conclusion, in our future judgement of the influence of heat treatment on coating properties, we can screen or determine to a certain extent whether the heat treatment process is reasonable or not by measuring only the macrotexture.

## 1. Introduction

In critical areas such as weaponry, transport and aerospace, a large number of metal components can trigger the service safety of materials due to exposure to the environment of high temperatures, wear and corrosion. Electrodeposition, as a surface treatment technology, is characterized by improved material properties and component service life [1,2,3,4,5,6,7]. In particular, electrodeposited chromium coatings are widely used in the military and aerospace fields such as gun barrels, gun tubes and airframes due to their good wear and corrosion resistance [8,9]. However, chromium atoms preferentially grow in specific directions during the electrodeposition process, forming a stronger texture and rendering the coating properties anisotropic. At the same time, hydrogen formed on the cathode due to hydrogen ion discharge enters the coating to form chromium hydride (CrH). Residual stress is present within the coating due to the crystallographic imbalance during electrodeposition. The presence of hydrogen, residual stress and the anisotropy of the coating reduces the material properties and service life, which in turn trigger material failure.

Several researchers have already found that the structure of the coating, the electrodeposition time, the temperature and the composition of plating solution all have an influence on the microstructure, which in turn forms or alters the residual stress and hydrogen content and influences other properties [10]. Surface treatment techniques such as electrodeposition are now widely applied, but subsequent heat treatment is still required to enable the coating to achieve the appropriate properties [11,12]. Heat treatment can effectively decrease the residual stress and hydrogen content, adjust the microstructure and obtain a better overall performance [13,14,15,16]. In previous studies, some researchers have carried out heat treatment after the plating of the chromium coating. When annealed at 1100 °C, the microhardness and wear resistance of the brush chromium coating improved, while microcracks reduced [17]. After heat treatment at 200–300 °C for 1 h, the microhardness of the chromium coating with organics as additives was significantly increased and the wear resistance was enhanced [18]. The grain size increased gradually with increasing heat treatment temperature and holding time, and the corrosion resistance of the electrodeposited chromium coating was significantly enhanced [19]. In arc ion chromium plating, mid-term ageing treatment at 1200 °C improved the oxidation resistance, and long-term ageing resulted in a decrease of properties [20].

It can be seen that the texture, hydrogen content and residual stress significantly influence the performance of a chromium coating. However, up to now, there are relatively few studies on how to regulate the texture and reduce the hydrogen content and residual stress of chromium coating. In this study, we identified the most appropriate process parameter to reduce hydrogen content and residual stress in the electrodeposited chromium coating on Cr–Ni–Mo–V steel substrate by a subsequent heat treatment process. The changes in the texture, microhardness and corrosion resistance were discussed and the microstructure–property relationship was established. The novelty was to provide a new approach to optimize the heat treatment process and to improve the service life of a chromium coating.

## 2. Experimental

### 2.1. Coating Preparation and Post Heat Treatment

Round Cr–Ni–Mo–V steel with a thickness of 10 mm and a diameter of 30 mm was used, and its composition is shown in Table 1. The anode was a lead plate whose distance from the cathode was 40 mm. The plating solution was composed of chromium trioxide (CrO_3_), chromium oxide (Cr_2_O_3_) and sulfuric acid (H_2_SO_4_). The substrate was pre-treated prior to chromium plating. For electroplating, the plating solution temperature and pH value were 65 °C and 5, respectively. The current density and the electrodeposition time were 35 A/dm^2^ and 11 h, respectively. After plating, the average thickness was about 38 μm.

**Figure 1:**
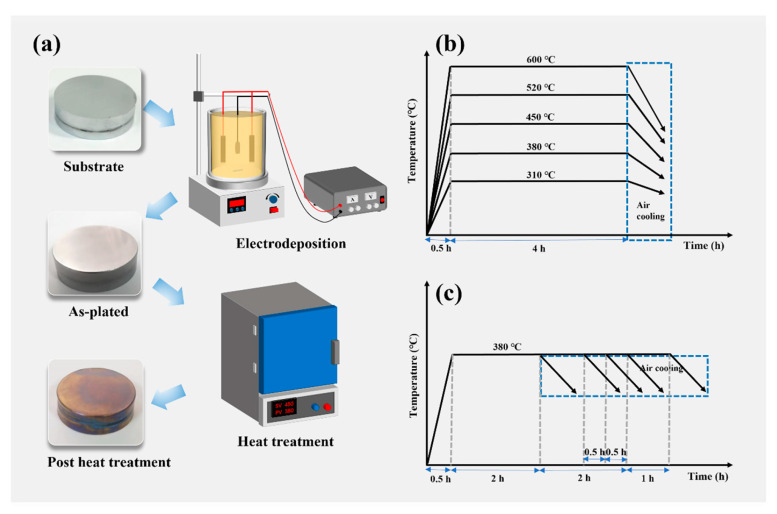
Schematic diagram of electrodeposition chromium coating and post heat treatment (**a**), different heat treatment temperature (**b**) and time (**c**) process curves.

Subsequently, the plated specimens were put in a muffle furnace (SX-G07103) for thermal treatment. The schematic diagram of electrodeposition chromium coating and post heat treatment is shown in Figure 1a. We combined the actual production conditions and process requirements of the factory to determine the post heat treatment temperature range of 38–520 °C and the range of heat treatment time of 2–5 h. The preferred heat treatment temperature was judged to be 380 °C by analyzing the residual stress and properties of the coating after heat treatment, and then experiments were carried out on the basis of 380 °C for different heat treatment times. The heat treatment process was as follows: (1) The specimens were heated in the furnace for 0.5 h. The specimens were held at the corresponding temperatures (310 °C, 380 °C, 450 °C, 520 °C, 600 °C) for 4 h, and the samples were taken out to be air-cooled. (2) We placed the samples in the furnace (room temperature: 25 °C) for 0.5 h, and then warmed them up in the furnace for 0.5 h, held them at 380 °C for the corresponding times (2 h, 3 h, 3.5 h, 4 h, 5 h), and the samples were removed and air-cooled. The heat treatment process curves are shown in Figure 1b,c.

### 2.2. Morphology and Microstructure Analysis

The morphology, dislocation density and microtexture were studied using a field emission scanning electron microscope (SUPRA 55, Carl-Zeiss, Jena, Germany) with an EBSD probe. The specimens were finely ground before testing, then mechanically polished and electrolytically polished. The crack density was counted by ImageJ. JS software.

**Figure 2:**
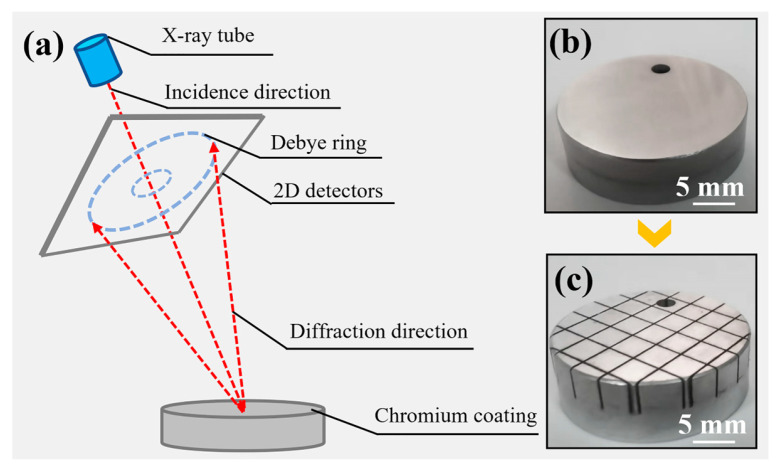
The test principle (**a**) of macrotexture, chromium plated specimen (**b**) and stress-free specimen (**c**).

The macrotexture tests were carried out using a portable X-ray diffractometer (μ-X360s, Pulstec, Japan). A Cr target was applied with an operating current of 1 mA and an operating voltage of 30 kV. The tested crystal plane was Cr (211) and the diffraction angle was 152.882°. The test principle is shown in Figure 2a. An X-ray diffractometer (D/MAX 2500 V L/PC, Rigaku, Tokyo, Japan) was used for the phase structure test. The test was performed with a Cu target, an operating current of 150 mA, a voltage of 40 kV, a scanning speed of 0.5 °/min, and a continuous scanning mode.

### 2.3. Hydrogen Content, Residual Stress, Microhardness and Corrosion Resistance Analysis

A diffusion hydrogen analyzer was utilized to test the hydrogen content. Residual stress test was carried out with a nano indenter (ST200, Fischer, German). The coating was heated to 800 °C to allow diffusible hydrogen to escape and the diffusible hydrogen was collected by thermal extraction. The indentation depth and load range were 2.0 μm and 0–150 gf, respectively. The presence of residual stress in the coating changed the slope of the load–depth curve [21]. At the same indentation depth, the load on the tensile stress specimen was less than the load in the stress-free condition. Therefore, the load difference between the sample to be tested and the stress-free specimen at the same depth reflected the magnitude of the residual stress. Stress-free specimens were obtained through cutting the coating surface to a mosaic grid of 4 × 4 × 4 mm^3^. The chromium-plated specimen and stress-free specimen are shown in Figure 2b,c. The residual stress σ and the difference in indentation load have the following relationship, as shown in Equation (1):

(1) $$\sigma =\frac{3}{2}\frac{(F_{0}-F_{S})H_{0}}{F_{S}}$$

where *F*_0_ is the indentation load (N) in the unstressed condition, *F_s_* is the indentation load (N) in the stressed condition, *σ* is the residual stress (MPa) and *H*_0_ is the microhardness (N/mm^2^) of the coating in the unstressed condition.

A digital microhardness tester was employed to test the microhardness. The test loading force was 100 gf and the loading time was 15 s. Princeton Versa STAT 3F electrochemical system workstation was used to evaluate the corrosion resistance of coatings. We carried out potentiodynamic polarization tests in 3.5% NaCl solution at room temperature (15 ± 1 °C). The three-electrode system consisted of a platinum electrode (counter electrode), a saturated calomel electrode (SCE, reference electrode) and a chromium coating (working electrode). Potentiodynamic polarization tests were carried out after 30 min of open circuit potential testing, and the polarization curves were scanned from −250 mV to +250 mV with respect to the open circuit potential at a rate of 0.5 mV/s.

## 3. Results

### 3.1. Morphology

**Figure 3:**
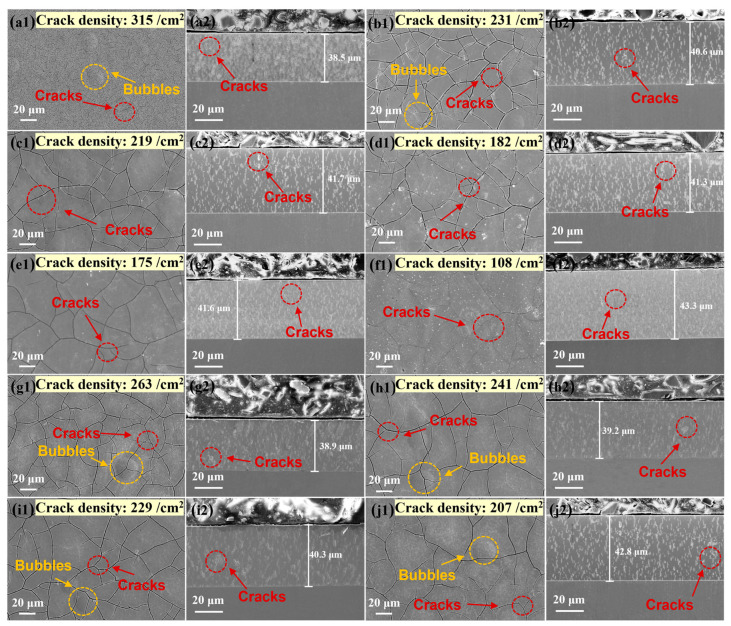
Surface morphology at different heat treatment temperatures and times. (**a1**) As-plated, (**b1**) 310 °C, 4 h, (**c1**) 380 °C, 4 h, (**d1**) 450 °C, 4 h, (**e1**) 520 °C, 4 h, (**f1**) 600 °C, 4 h, (**g1**) 380 °C, 2 h, (**h1**) 380 °C, 3 h, (**i1**) 380 °C, 3.5 h, (**j1**) 380 °C, 5 h. Cross-section morphology at different heat treatment temperatures and times. (**a2**) As-plated, (**b2**) 310 °C, 4 h, (**c2**) 380 °C, 4 h, (**d2**) 450 °C, 4 h, (**e2**) 520 °C, 4 h, (**f2**) 600 °C, 4 h, (**g2**) 380 °C, 2 h, (**h2**) 380 °C, 3 h, (**i2**) 380 °C, 3.5 h, (**j2**) 380 °C, 5 h.

Hydrogen penetration and residual stress can cause the formation and expansion of microcracks, which have a significant impact on the corrosion and abrasion resistance of the coating. The coating morphology and thickness will be changed after heat treatment. At a heat treatment temperature of 600 °C, the coating thickness (43.3 μm) increased by 4.8 μm compared to the as-plated state (38.5 μm), as seen in Figure 3. This was mainly attributed to the oxidization of the coating. Meanwhile, the crack density of the coating surface decreased to different degrees with the increase of the heat treatment temperature and time. Among them, the crack density was 315/cm^2^, 231/cm^2^, 219/cm^2^, 182/cm^2^, 175/cm^2^ and 108/cm^2^ in the as-plated state and at different heat treatment temperatures (310 °C, 380 °C, 450 °C, 520 °C and 600 °C), respectively. The reduction in crack density arose from two main sources. For one thing, residual tensile stresses generally provided the energy for crack extension, and heat treatment could reduce the residual tensile stresses, which in turn decreased the tendency of crack formation and extension. For another matter, under certain heat treatment conditions, the coating may form oxide films to close the cracks [22]. This improved the densification of the coating, which in turn enhanced the corrosion resistance [23]. In addition, the crack depths did not vary much during different heat treatment processes, and the depths generally ranged from 3.5 μm to 4.6 μm.

### 3.2. Texture

#### 3.2.1. Macrotexture

**Figure 4:**
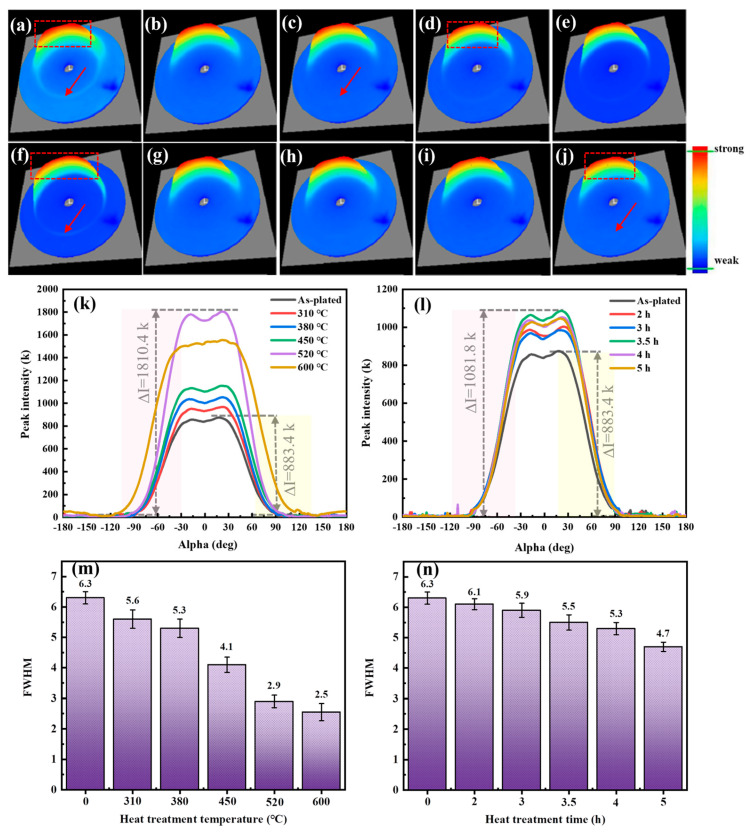
Debye ring at different heat treatment temperatures and times: (**a**) As-plated, (**b**) 310 °C, 4 h, (**c**) 380 °C, 4 h, (**d**) 450 °C, 4 h, (**e**) 520 °C, 4 h, (**f**) 600 °C, 4 h, (**g**) 380 °C, 2 h, (**h**) 380 °C, 3 h, (**i**) 380 °C, 3.5 h, (**j**) 380 °C, 5 h. Variation of Debye ring diffraction peak intensity: (**k**) differences in heat treatment temperature and (**l**) differences in heat treatment time. Variation of FWHM: (**m**) differences in heat treatment temperature and (**n**) differences in heat treatment time.

Figure 4a,j represent the Debye ring at different heat treatment temperatures and times. Figure 4k,l show the variation of Debye ring diffraction peak intensity. The color corresponded to the peak intensity and the position corresponded to the alpha. Red and blue colors represented high and low intensity, respectively. When the material was isotropic, there should have been no large fluctuations in the intensity. However, the Debye ring had a large absence in either state, demonstrating a preferred orientation. The change in the extreme difference in peak intensity was 883.4 k in the as-plated state. With the increase of heat treatment temperature and time, the diffraction peak intensity difference exhibited a tendency of increasing and subsequently decreasing. The diffraction peak intensity difference was relatively high at the heat treatment temperature of 520 °C and the time of 3.5 h, which was 1810.4 k and 1081.8 k, respectively. Figure 4m,n indicate the FWHM for different heat treatment temperatures and times, respectively. The FWHM gradually decreased with heat treatment. The tendency of the FWHM to decrease with the heat treatment temperature was greater than with the heat treatment time. This was mainly due to the grain coarsening and the decrease in residual stresses during the eat treatment [24].

**Figure 5:**
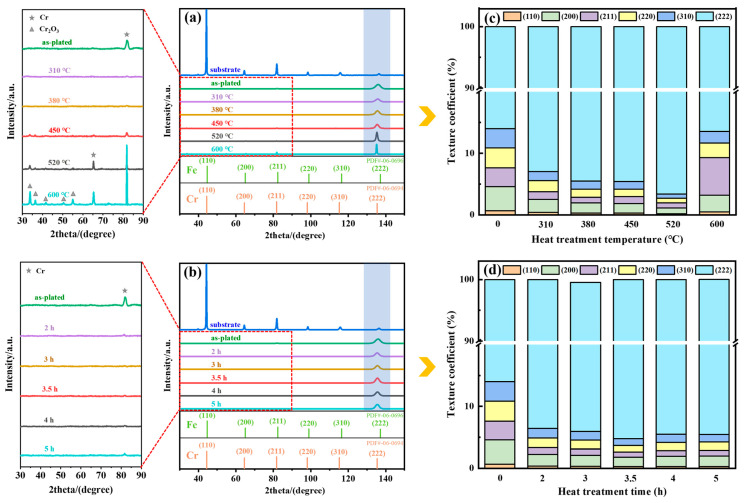
XRD patterns. Differences in heat treatment temperature (**a**) and time (**b**). Bar graphs of relative texture coefficients. Differences in heat treatment temperature (**c**) and time (**d**).

Then, the crystal structure of the substrate and the chromium coating were analyzed. The XRD patterns at different heat treatment temperatures and times are seen in Figure 5a,b. The strongest peak of the coating of the standard and measured diffraction spectrum appeared in (110) and (222), respectively. Cr_2_O_3_ was detected at heat treatment temperatures of 450 °C and above, indicating oxidation of the coating. The intensity of the diffraction peak in the (222) progressively increased by increasing the heat treatment temperature, indicating a variation in the texture [25]. The diffraction peak intensities of the (222) crystal plane changed less with the increase in heat treatment time. In order to calculate the variations in the texture content of the chromium coating, the degree of preferred orientation was expressed by applying the relative texture coefficient RTC_(hkl)_, as shown in Equation (2) [26].

(2) $${RTC}_{\left(hkl\right)}=I_{\left(hkl\right)}/I_{0\left(hkl\right)}/\sum_{i=1}^{n}I_{\left(hikili\right)}/I_{0\left(hikili\right)}\times 100\%$$

where I_(hkl)_ and I_0(hkl)_ are the intensity (k) of the chromium coating and the standard chromium powder at (hkl).

The relative texture coefficients for different heat treatment conditions are seen in Table 2. Figure 5c,d depict the change of the relative texture coefficients for different heat treatment temperatures and times. When the relative texture coefficient of a plane is above the average coefficient value (16.7%), the crystal growth shows anisotropy [27]. The texture coefficient of the coating at the (222) crystal plane was consistently greater than 85% with heat treatment. With the increase of heat treatment temperature, the texture of (222) increased and subsequently decreased, and it was the highest when the temperature was 520 ℃. The change in the texture of (222) was not significant by increasing heat treatment time.

#### 3.2.2. Microtexture

**Figure 6:**
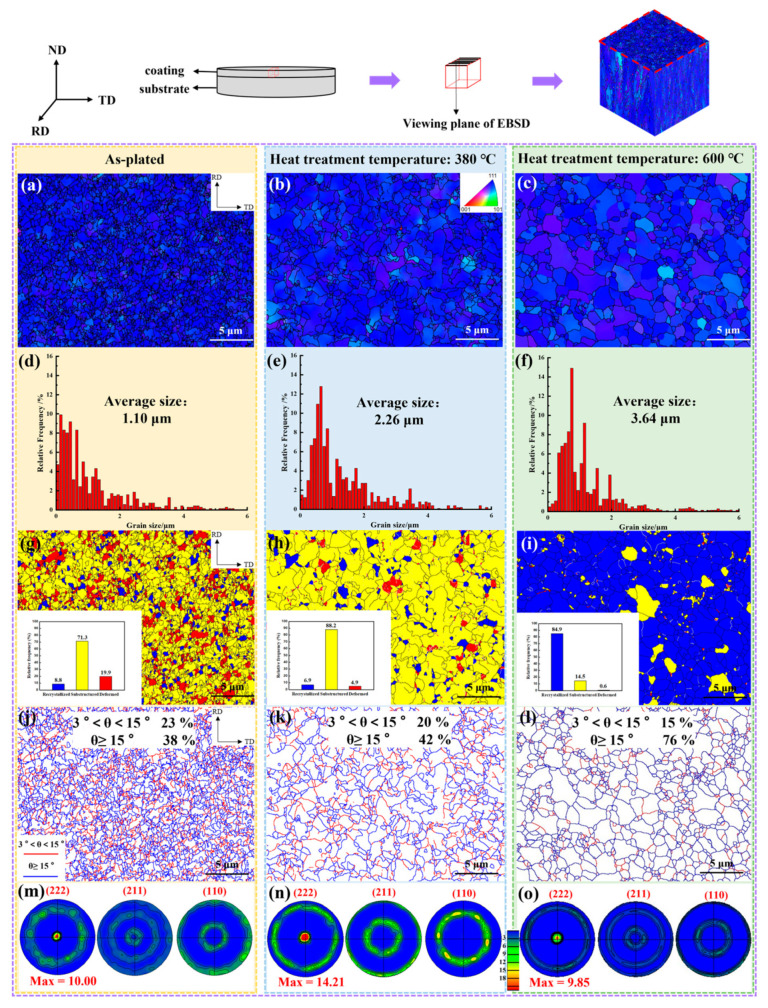
Surface grain orientation distribution. As-plated (**a**), heat treatment temperatures of 380 °C (**b**) and 600 °C (**c**). Grain size distribution. As-plated (**d**), heat treatment temperatures of 380 °C (**e**) and 600 °C (**f**). Recrystallization distribution. As-plated (**g**), heat treatment temperatures of 380 °C (**h**) and 600 °C (**i**). Grain boundary distribution. As-plated (**j**), heat treatment temperatures of 380 °C (**k**) and 600 °C (**l**). Pole figures. As-plated (**m**), heat treatment temperatures of 380 °C (**n**) and 600 °C (**o**).

**Figure 7:**
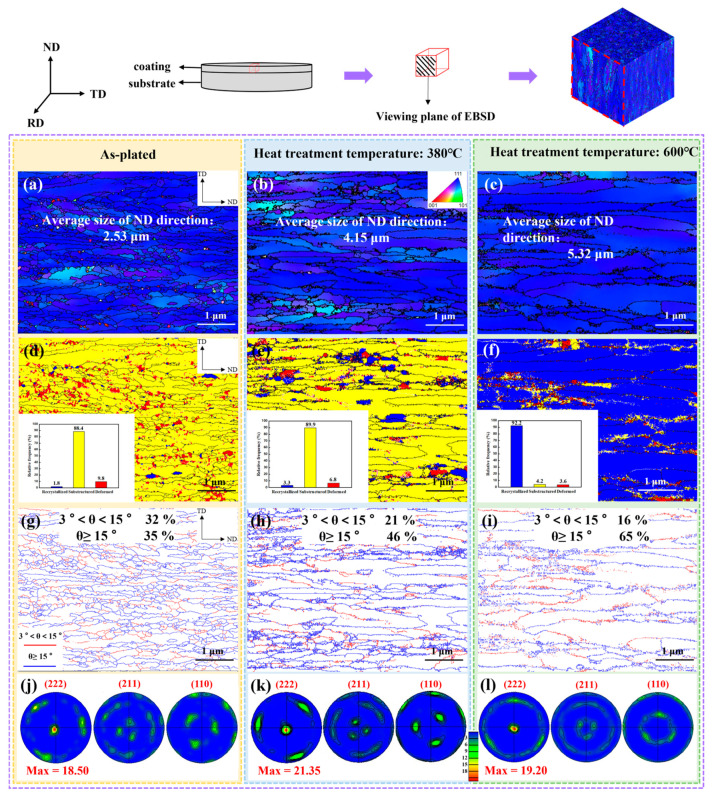
Cross-section grain orientation distribution. As-plated (**a**), heat treatment temperatures of 380 °C (**b**) and 600 °C (**c**). Recrystallization distribution. As-plated (**d**), heat treatment temperatures of 380 °C (**e**) and 600 °C (**f**). Grain boundary distribution. As-plated (**g**), heat treatment temperatures of 380 °C (**h**) and 600 °C (**i**). Pole figures. As-plated (**j**), heat treatment temperatures of 380 °C (**k**) and 600 °C (**l**).

The relative texture coefficient revealed that the influence of the heat treatment time on the texture was relatively low, therefore the microtexture was analyzed mainly for the as-plated state and the 380 °C and 600 °C heat treatment temperatures. Figure 6a–c and Figure 7a–c represent the surface and cross-section grain orientation distribution, respectively. The similarity of the grain colors demonstrated that the texture was consistently stronger in several states.

To identify the type of texture, the texture was counted by means of pole figures in Figure 6m-o and Figure 7j–l. Red and blue colors represented high and low intensity, respectively. The fiber texture was found in the chromium coating and the type of texture was not altered by heat treatment [28]. The values of the pole density of the (222) plane on the surface of the coating were 10.00, 14.21 and 9.85 for increasing heat treatment temperatures. The trend of the texture was in accordance with the results of the calculation of the relative texture coefficient RTC_(hkl)_. Meanwhile, the texture evolution pattern of the coating cross-section (222) crystal plane was consistent with that of the surface.

### 3.3. Microstructure

Figure 6d–f present the surface grain distribution at different heat treatment temperatures. With the increase of temperature, the grain size gradually grew owing to recrystallization. The grain sizes of the coating surface were 1.10 μm, 2.26 μm and 3.64 μm in the as-plated state and at 380 °C and at 600 °C heat treatment temperatures, respectively, and the cross-section grain sizes were 2.53 μm, 4.15 μm and 5.32 μm.

Combining the coating surface and cross-section recrystallization distribution diagrams in Figure 6g–i and Figure 7d–f, the chromium coating was predominantly columnar crystals in the as-plated state [29,30]. After heat treatment to 380 °C, the proportion of the substructure was elevated and the grain boundaries in the coating cross-section tended to straighten out. Consequently, during this period, the grains mainly underwent a recovery process [20]. After heat treatment to 600 °C, the proportion of the recrystallization microstructure of the coating surface increased from 6.9% to 84.9%, and the grains also had a tendency to transform to equiaxed grains. This can indicate that the grains have gone through the recrystallization process [31].

The surface and cross-section grain boundary distribution diagrams are shown in Figure 6j–l and Figure 7g–i. Boundaries between 3° and 15° were defined as small angle boundaries, indicated by the red line, and boundaries larger than 15° were large angle boundaries, represented by the blue line. As the heat treatment temperature increased, the small angle grain boundary decreased and the large angle grain boundary increased significantly. The change was particularly significant at 600 °C. It was attributed to the significant reduction of dislocations during rearrangement or annihilation during recrystallization. The formation of numerous new grain boundaries facilitated the release of residual stresses [32].

**Figure 8:**
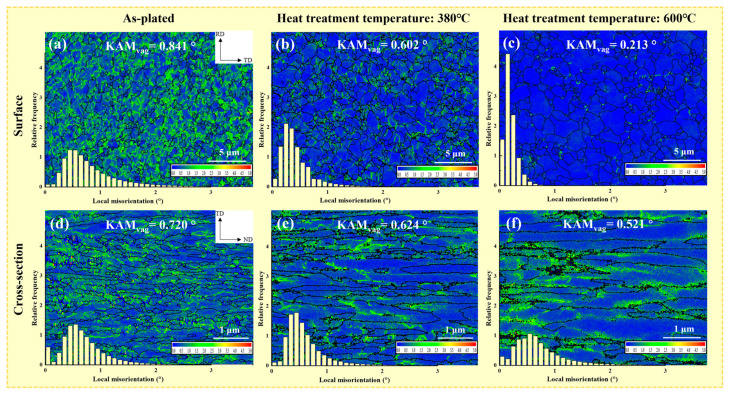
Surface local misorientation distribution for as-plated (**a**) and heat treatment temperatures of 380 °C (**b**) and 600 °C (**c**). Cross-section local misorientation distribution for as-plated (**d**) and heat treatment temperatures of 380 °C (**e**) and 600 °C (**f**).

Dislocation density, i.e., defects, affected the residual stress, microhardness and other properties of the chromium coating. Figure 8 shows the local misorientation distribution of coating surface and cross-section in the as-plated state and at 380 °C and 600 °C heat treatment temperatures. The average local misorientation on the coating surface was 0.841°, 0.602 °C and 0.213 °C with increasing heat treatment temperature and 0.720 °C, 0.624 °C, and 0.521 °C in cross section, respectively. The geometric necessary dislocation density (*ρ_GND_*) was calculated from the average local misorientation (*θ_KAM_*) as shown in Equation (3) [29]. The calculations are shown in Table 3. The dislocation density on the surface of the coating tended to decrease more than the cross-section with increasing heat treatment temperature. The dislocation density decreased slowly in the recovery process and rapidly in the recrystallization process at the coating surface.

(3) $$\rho_{GND}≅2\theta_{KAM}/bd$$

where the Bragg vector length *b* of the chromium metal and the EBSD scan step *d* are 0.250 nm and 0.05 μm, respectively [33].

### 3.4. Hydrogen Content

**Figure 9:**
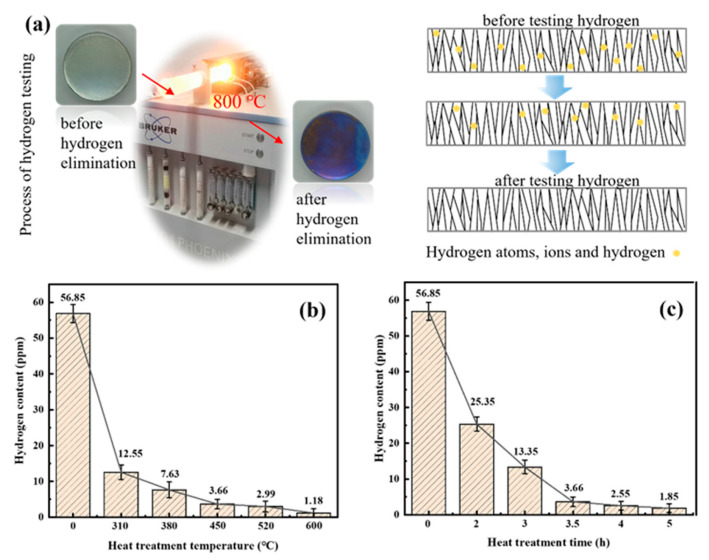
Schematic diagram of hydrogen content test (**a**) and variation of hydrogen content curves for differences in heat treatment temperature (**b**) and time (**c**).

During electrodeposition, hydrogen may trigger volume shrinkage of the chromium coating, causing residual stress and even cracking. Therefore, knowing how to reduce the hydrogen content was essential to improve this property of the coating. The test principle was to dehydrogenate the coating by diffusion of hydrogen from the coating to the surface microcracks through molecular thermal movement, as shown in Figure 9a. The hydrogen content variation curves at different heat treatment temperatures and times are seen in Figure 9b–c. At different heat treatment temperatures, the hydrogen content decreased at a faster rate than the heat treatment time.

The hydrogen content was 7.63 ppm and 1.18 ppm at the heat treatment temperatures of 380 °C and 600 °C, respectively, with 86% and 98% reductions compared to the as-plated state. When the heat treatment temperature was higher than 450 °C and the time was longer than 4 h, the decreasing trend of hydrogen content was slower. Therefore, considering the reduction of production costs and the hazards of hydrogen embrittlement, the heat treatment temperature range for hydrogen removal was between 380 °C and 450 °C, and the heat treatment time was between 3.5 h and 4 h.

### 3.5. Residual Stress

**Figure 10:**
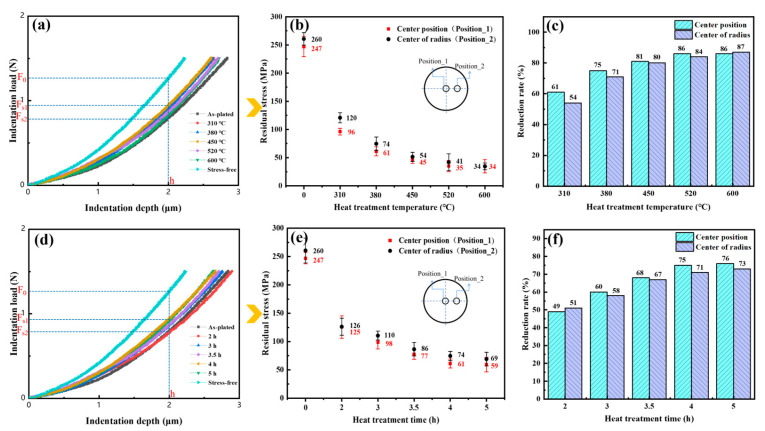
Load-depth curves (**a**), residual stresses (**b**) and reduction rates (**c**) for different heat treatment temperatures. Load-depth curves (**d**), residual stresses (**e**) and reduction rates (**f**) for different heat treatment times.

The load-depth curves of different heat treatment temperatures and times are shown in Figure 10a,d. The variation of residual stresses at different temperature and time heat treatments is shown in Figure 10b,e. The residual tensile stress at the circle center of the as-plated state was 247 MPa. After heat treatment, the residual tensile stress decreased, which was also reflected in other post heat treatments [25]. The reduction rate of the residual stress after different treatment temperatures and times is found in Figure 10c,f. The residual stress reduction rate of the coating was 75% at the heat treatment temperature of 380 °C. Above the 380 °C heat treatment temperature and the 4 h heat treatment time, the variation of the stress reduction rate was relatively slow.

### 3.6. Microhardness

**Figure 11:**
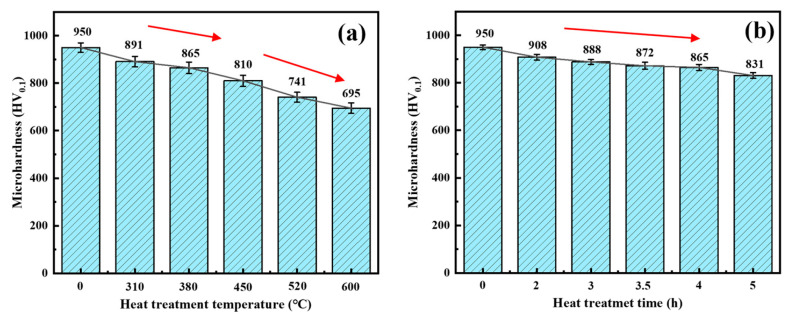
Variation of surface microhardness curves for differences in heat treatment temperature (**a**) and differences in time (**b**).

The microhardness of coatings was generally associated with dislocation density, grain size, grain orientation and residual stress. The microhardness for different heat treatment temperatures and times is shown in Figure 11a,b. It was observed that the microhardness decreased significantly with heat treatment. Since the grain size was in the micrometer range (see Figure 6d–f) and based on the Hall–Petch relationship (see Equation (4) [34]), the lessening in microhardness was mainly due to grain coarsening under the enhancement of the (222) texture. Meanwhile, the reduction in the microhardness was also related to the reduction of residual stress (see Figure 10b,e) [35].

(4) $$H_{V}=H_{0}+k_{H}D^{-1/2}$$

where *H_0_* and *k_H_* are constants, and *D* and *H_V_* are grain size and microhardness, respectively.

The microhardness in the as-plated state was 950 HV. The microhardness decreased relatively slowly with increasing heat treatment time. With the increase of heat treatment temperature, the microhardness was divided into two stages. When the temperature was below 380 °C, the microhardness decreased at a slow rate, and the microhardness was 865 HV at the heat treatment temperature of 380 °C. When the temperature reached 600 °C, the microhardness decreased rapidly and was accompanied by a microhardness of 695 HV. The reduction in dislocation density led to a decrease in the intersection of dislocations during movement, causing less dislocation pile-up and thus a diminishment of microhardness [36]. Although the texture of (222) decreased at the heat treatment temperature of 600 °C, the microhardness still tended to decrease rapidly due to the significant decrease in the dislocation density at this stage. Therefore, the variation of microhardness can be reflected according to the trend of decreasing dislocation density at the heat treatment temperatures of 380 °C and 600 °C (see Figure 8a–c).

### 3.7. Corrosion Resistance

**Figure 12:**
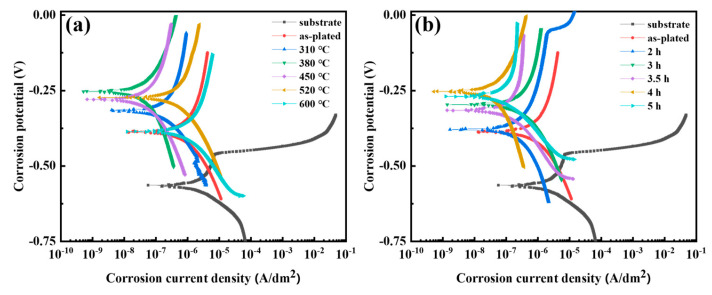
Potentiodynamic polarization curves in 3.5 wt.% NaCl solution for differences in heat treatment temperature (**a**) and differences in time (**b**).

The potentiodynamic polarization curves at different heat treatment temperatures and times are shown in Figure 12a,b. The corrosion potential E_corr_ and corrosion current density i_corr_ are shown in Table 4. The corrosion potentials E_corr_ of the substrate as well as the coating in the as-plated state were −571.6 mV and −389.2 mV, respectively. As the heat treatment temperature and time increased, the corrosion potential E_corr_ of the coating was more positive at the heat treatment temperature and time of 380 °C and 4 h, about −253.2 mV. This indicated that the coating under the process exhibited a higher degree of nobility. The corrosion current densities i_corr_ of the substrate and as-plated state coating were, respectively, 3.654 × 10^−3^ A/dm^2^ and 1.895 × 10^−5^ A/dm^2^. Under various heat treatment conditions, the corrosion current density of the coating was lower (approximately 1.798 × 10^−6^ A/dm^2^) at the heat treatment temperature and time of 380 °C and 4 h. In sum, the heat treatment temperature and time of 380 °C and 4 h of the coating showed higher corrosion potential and lower corrosion current density, which provided more favorable corrosion resistance [37].

## 4. Discussion

### 4.1. Evolution of Texture

Hydrogen adsorption during electrodeposition results in differences of the growth rate at every crystal plane, which consequently affects the surface energy and hence the formation of a preferred orientation [38]. When the (hkl) index is high, the more intensified the inhibition of crystal growth; this is due to the fact that at this point, the chemical activity is stronger and the adsorption of hydrogen is higher [39]. Consequently, the stronger texture in the as-plated state of the (222) plane was due to the higher hydrogen content in the coating, which led to an increased inhibition of grain growth of the (211) plane.

The development of the coating texture after heat treatment is mainly impacted by grain growth drivers such as surface energy and strain energy [40]. The transformation of the texture is often the result of a combination of surface and strain energies at the crystal plane [41]. Thus, the orientated grains of (222) with the lowest surface energy or other orientations with lower strain energy may grow preferentially during the heat treatment process. At the heat treatment temperature of 380 °C, the coating mainly recovered, with the more strongly orientated grains gradually subsuming the less strongly orientated grains. This may be due to the surface energy dominance before the temperature of 380 °C and the lower surface energy of the most densely stacked (222) crystal plane, hence the enhanced (222) orientation. After the 600 °C heat treatment, recrystallization occurred and the coating formed other preferred orientation grains. This might be due to the combined effect of surface and strain energies, resulting in a relative weakening of the (222) orientation and an increase in the strength of the (211) crystal plane texture. This was consistent with the trend of the change in the (222) plane texture of the metal coatings during the annealing process [41,42].

### 4.2. Influence of Microstructure on Hydrogen Content

The variation of hydrogen content of the coating during heat treatment may be related to the crystal orientation. The differences in electrochemical activity resulted in different rates of grain growth on the crystal plane of the chromium coating. According to the relationship between the crystal plane index and hydrogen adsorption, hydrogen is more likely to be adsorbed on planes with a higher crystal plane index [39]. Therefore, the chromium coating (211) crystal plane exhibited a better adsorption capacity for hydrogen than the (222) crystal plane. The diffusion of hydrogen from the (222) crystal plane was more likely to occur.

At lower heat treatment temperatures, the (222) crystal plane texture was gradually enhanced with increasing heat treatment temperature. The adsorption capacity of hydrogen content on the (222) plane was weakened, creating favorable conditions for hydrogen diffusion. When the temperature reached 600 °C, the (222) crystal plane texture was weakened and the (211) plane texture was strengthened. Hydrogen would preferentially adsorb on the (211) crystal plane, so that the diffusion rate of hydrogen decreased. This explained the reason for the slower decrease of hydrogen content when the heat treatment temperature reached 600 °C.

### 4.3. Influence of Microstructure on Residual Stress

The reduction of residual stress was mainly associated with the following two aspects. For one thing, the formation of residual stress may be connected to grain size, dislocation density, hydrogen content and grain orientation. One of the factors contributing to the formation of tensile residual stress could be the consequence of mismatched grain adaptation boundaries during grain merging [43]. A lower grain growth depression led to lower nucleation rates which in turn reduced tensile residual stresses. With the grain coarsening under the (222) plane texture enhancement, the tensile stress in the coating diminished (see Figure 6d–f). When the dislocation density was higher, the more lattice distortion was caused and the higher the residual stress. Therefore, the reduction of stress was also attributed to the decline in dislocation density after heat treatment. Furthermore, the hydrogen content diminished gradually with heat treatment. The decrease of the hydrogen of lattice induced by the enhancement of the (222) texture led to a decrease in the stress. For heat treatment temperatures up to 600 °C, however, the texture of (222) was weakened. However, under the influence of increased grain size and the significant decrease in dislocation density brought about by the higher heat treatment temperature, the coating stress decreased further, but the downward trend decreased.

For another matter, the reduction in residual stress may be associated with the yield strength of the material. The yield strength was reduced with higher heat treatment temperatures, and when the yield strength of a material fell below a certain value, the material would equilibrate the residual stress through local plastic deformation [44]. At the same time, the creep generated during the heat treatment also led to stress release.

### 4.4. Influence of Microstructure on Corrosion Resistance

The corrosion resistance of the coating was related to crystal orientation, microcracks, dislocations and grain size. In a corrosive environment, there were certain differences in the corrosion rates of grains on different crystal planes. For densely arranged atomic planes (e.g., the (222) crystal plane), the high binding energy of the atoms renders the surface more resistant to dissolution [45]. As the heat treatment temperature increased, the texture strength of the (222) plane first increased and then decreased (as shown in Figure 5c), which led to a decrease in the corrosion resistance at higher heat treatment temperatures. Since the effect of heat treatment time on the texture of the (222) plane was relatively low, this was not the main factor determining the variation of the corrosion resistance of the coatings at different heat treatment times.

The number of microcracks on the coating surface decreased significantly with increasing heat treatment temperature (as shown in Figure 3). This reduced the opportunity for chloride ions to penetrate into the substrate through the cracks, which explains the gradual increase in corrosion resistance at heat treatment temperatures below 450 °C. The corrosion rate of the chromium coating was also correlated with the dislocation density. When there were more defects, there was a greater amount of dissolution of the coating at the initiation of anodic polarization. As the dislocation density gradually decreased with heat treatment (as shown in Figure 8), the corrosion resistance generally showed an increasing trend. In addition, materials with smaller grain sizes tended to have better corrosion resistance [46]. Therefore, excessive grain size caused by high heat treatment temperature and time (as shown in Figure 6c) was detrimental to the corrosion resistance of the coating.

### 4.5. The Microstructure–Property Relationship

**Figure 13:**
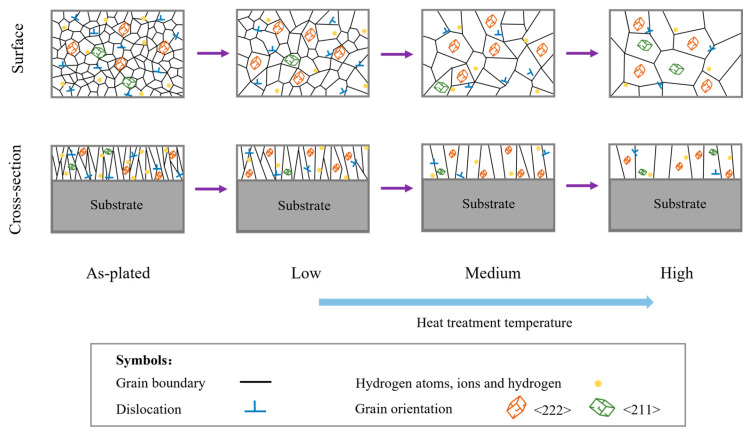
Schematic diagram of the hydrogen and microstructure evolution during the heat treatment process.

It was necessary to regulate the microstructure and properties of the chromium coating by selecting a suitable heat treatment process. The schematic diagram of the hydrogen and microstructure evolution during the heat treatment process is shown in Figure 13. The crack density, residual stress and properties at different heat treatment conditions are shown in Table 5. When choosing the heat treatment temperature, it was noted that the decreasing trend of hydrogen content and residual stress gradually decreased when the heat treatment temperature was above 380 °C. However, the microhardness and corrosion resistance decreased significantly at temperatures above 380 °C. At 380 °C, the crack density and dislocation density were comparatively low and the grain size was not too large. Although the texture on the (222) crystal plane was stronger at this temperature, the heat treatment temperature of 380 °C should be selected in consideration of the comprehensive performance of the coating.

With heat treatment times longer than 4 h, not only did the microhardness and corrosion resistance of the coating decrease further, but also the decreasing tendency of the stress and hydrogen content was relatively low. At the same time, the texture of the coating did not weaken. In summary, considering the microstructure, hydrogen content, residual stress, microhardness and corrosion resistance, the optimum heat treatment process was a heat treatment temperature of 380 °C and a time of 4 h. Overall, when the heat treatment temperature fell below 600 °C, the increase in the strength of the (222) texture brought about a decrease of hydrogen content, residual stress and microhardness and an increase of corrosion resistance. Therefore, when we judge the variation of coating properties caused by heat treatment in the future, we can screen or determine whether the heat treatment process is reasonable by measuring only the macrotexture.

## 5. Conclusions

(1) As the heat treatment temperature increased from 310 °C to 600 °C, the crack density decreased from 231/cm^2^ to 108/cm^2^. The grain size and the number of large angle boundaries subsequently increased and the dislocation density decreased.
(2) The hydrogen adsorption in the as-plated state caused the coating to form a strong fiber texture on the (222) plane, and its texture strength increased and then decreased with increasing heat treatment temperature. When the heat treatment temperature was below 600 °C, the increase in the strength of the (222) texture promoted a decrease in the hydrogen content, residual stress and microhardness and an increase in the corrosion resistance. The heat treatment time had less effect on the crack density and texture strength.
(3) The preferable heat treatment temperature and time were 380 °C and 4 h, respectively. In this process, the crack density, dislocation density, grain size and pole density value of the (222) plane were 219/cm^2^, 1.68 × 10^15^ m^−2^, 2.26 μm and 14.21, and the proportion of large angle boundaries and recovery microstructure were 42% and 88.2%, respectively. The hydrogen content, residual stress, microhardness and corrosion current density were found to be 7.63 ppm, 61 MPa, 865 HV and 1.798 × 10^−6^ A/dm^2^, respectively. The reduction rates of the residual stress and hydrogen content compared to the as-plated state were 86% and 75%.
(4) In our future research on the changing laws of coating properties caused by heat treatment, we can judge to a certain extent whether the heat treatment process is reasonable or not by only measuring the macrotexture.

## Figures and Tables

**Table 1:** Substrate composition (wt.%).

Element	Fe	C	Si	Cr	Ni	Mo	V	W	Nb
Content	Bal.	0.27~0.31	≤0.40	2.75~3.05	1.15~1.30	1.70~1.85	0.40~0.55	0.45~0.65	0.01~0.04

**Table 2:** Relative texture coefficients (RTC_(hkl)_) at different heat treatment conditions.

Temperature (°C)	Time	RTC_(hkl)_/%					
	(h)	(110)	(200)	(211)	(220)	(310)	(222)
0	0	0.7	3.9	3.0	3.2	3.2	86.0
310	4	0.4	2.1	1.2	1.8	1.5	93.0
380	4	0.5	1.9	1.1	1.5	1.5	94.5
450	4	0.3	1.5	1.2	1.2	1.2	94.6
520	4	0.2	0.8	0.7	0.6	0.7	97.0
600	4	0.5	2.7	6.1	2.4	1.9	86.4
380	2	0.4	1.9	1.1	1.5	1.6	93.5
380	3	0.2	1.8	1.2	1.5	1.4	93.6
380	3.5	0.3	1.5	0.8	1.1	1.1	95.2
380	5	0.3	1.7	0.9	1.3	1.2	94.6

**Table 3:** Geometric necessary dislocation density (ρ_GND_) at different heat treatment conditions.

Temperature (°C)	Time (h)	Grain Direction	*ρ_GND_* (10^15^ m^−2^)
0	0	surface	2.35
380	4	surface	1.68
600	4	surface	0.59
0	0	cross-section	2.01
380	4	cross-section	1.74
600	4	cross-section	1.45

**Table 4:** Corrosion potential, E_corr_, and corrosion current density, i_corr_, for different heat treatment conditions.

Temperature (°C)	Time (h)	E_corr_ (mV)	i_corr_ (A/dm^2^)	Temperature (°C)	Time (h)	E_corr_ (mV)	i_corr_ (A/dm^2^)
0	0	−389.2 ± 11.6	1.895 × 10^−5^ ± 0.112 × 10^−5^	380	2	−379.2 ± 13.6	2.052 × 10^−6^ ± 0.162 × 10^−6^
310	4	−314.3 ± 9.8	1.634 × 10^−5^ ± 0.101 × 10^−5^	380	3	−296.5 ± 12.3	1.936 × 10^−6^ ± 0.161× 10^−6^
380	4	−253.2 ± 12.1	1.798 × 10^−6^ ± 0.125 × 10^−6^	380	3.5	−314.9 ± 9.9	1.855 × 10^−6^ ± 0.108 × 10^−6^
450	4	−279.1 ± 10.2	1.835 × 10^−6^ ± 0.124 × 10^−6^	380	5	−269.2 ± 11.8	1.837 × 10^−6^ ± 0.121 × 10^−6^
520	4	−272.9 ± 10.2	1.756 × 10^−5^ ± 0.135 × 10^−5^	substrate	substrate	−571.6 ± 13.5	3.654 × 10^−3^ ± 0.106 × 10^−3^
600	4	−386.2 ± 11.9	1.982 × 10^−5^ ± 0.215 × 10^−5^				

**Table 5:** Crack density, residual stress and properties for different heat treatment conditions.

Temperature (°C)	Time (h)	Crack Density (/cm^2^)	Residual Stress (MPa)	Hydrogen Content (ppm)	Microhardness (HV_0.1_)	i_corr_ (A/dm^2^)
0	0	315	247	56.85	950	1.895 × 10^−5^
310	4	231	96	12.55	891	1.634 × 10^−5^
380	4	219	61	7.63	865	1.798 × 10^−6^
450	4	182	45	3.66	810	1.835 × 10^−6^
520	4	175	35	2.99	741	1.756 × 10^−5^
600	4	108	34	1.18	695	1.982 × 10^−5^
380	2	263	125	25.35	908	2.052 × 10^−6^
380	3	241	98	13.35	888	1.936 × 10^−6^
380	3.5	229	77	3.66	872	1.855 × 10^−6^
380	5	207	59	1.85	831	1.837 × 10^−6^

## Data Availability

All source data may be obtained from the corresponding author.
